# (Ex-)breast cancer patients with (pre-existing) symptoms of anxiety and/or depression experience higher barriers to contact health care providers during the COVID-19 pandemic

**DOI:** 10.1007/s10549-021-06112-y

**Published:** 2021-02-18

**Authors:** Dieuwke R. Mink van der Molen, Claudia A. Bargon, Marilot C. T. Batenburg, Roxanne Gal, Danny A. Young-Afat, Lilianne E. van Stam, Iris E. van Dam, Femke van der Leij, Inge O. Baas, Miranda F. Ernst, Wiesje Maarse, Nieke Vermulst, Ernst J. P. Schoenmaeckers, Thijs van Dalen, Rhodé M. Bijlsma, Annemiek Doeksen, Helena M. Verkooijen, UMBRELLA study group

**Affiliations:** 1 Division of Imaging and Oncology, University Medical Center Utrecht, Heidelberglaan, Utrecht, The Netherlands; 2 Department of Surgery, St. Antonius Hospital, Soestwetering, Utrecht, The Netherlands; 3 Department of Plastic, Reconstructive and Hand Surgery, Amsterdam University Medical Center, Location VUmc, Amsterdam, The Netherlands; 4 Department of Radiation Oncology, University Medical Center Utrecht, Cancer Center, Utrecht, The Netherlands; 5 Department of Medical Oncology, University Medical Center Utrecht, Cancer Center, Utrecht, The Netherlands; 6 Department of Surgery, Alexander Monro Clinics, Bilthoven, The Netherlands; 7 Department of Plastic, Reconstructive and Hand Surgery, University Medical Center Utrecht, Utrecht, The Netherlands; 8 Department of Surgery, Rivierenland Hospital, Tiel, The Netherlands; 9 Department of Surgery, Meander Medisch Centrum, Amersfoort, The Netherlands; 10 Department of Surgery, Diakonessenhuis Utrecht, Utrecht, The Netherlands; 11 Utrecht University, Heidelberglaan, Utrecht, The Netherlands

**Keywords:** Breast cancer, COVID-19, Depression, Anxiety, UMBRELLA, Patient-reported outcomes

## Abstract

**Purpose:**

To identify factors associated with (perceived) access to health care among (ex-)breast cancer patients during the COVID-19 pandemic.

**Methods:**

Cross-sectional study within a large prospective, multicenter cohort of (ex-)breast cancer patients, i.e., UMBRELLA. All participants enrolled in the UMBRELLA cohort between October 2013 and April 2020 were sent a COVID-19-specific survey, including the Hospital Anxiety and Depression Scale (HADS) questionnaire.

**Results:**

In total, 1051 (66.0%) participants completed the survey. During COVID-19, 284 (27.0%) participants reported clinically relevant increased levels of anxiety and/or depression, i.e., total HADS score ≥ 12. Participants with anxiety and/or depression reported statistically significant higher barriers to contact their general practitioner (47.5% vs. 25.0%, resp.) and breast cancer physicians (26.8% vs. 11.2%, resp.) compared to participants without these symptoms. In addition, a higher proportion of participants with anxiety and/or depression reported that their current treatment or (after)care was affected by COVID-19 compared to those without these symptoms (32.7% vs. 20.5%, resp.). Factors independently associated with symptoms of anxiety and/or depression during COVID-19 were pre-existent anxiety (OR 6.1, 95% CI 4.1–9.2) or depression (OR 6.0, 95% CI 3.5–10.2).

**Conclusion:**

During the COVID-19 pandemic, (ex-)breast cancer patients with symptoms of anxiety and/or depression experience higher barriers to contact health care providers. Also, they more often report that their health care was affected by COVID-19. Risk factors for anxiety and/or depression during COVID-19 are pre-existent symptoms of anxiety or depression. Extra attention—including mental health support—is needed for this group.

## Introduction

The COVID-19 pandemic is having a major impact on global health. During the early months of the COVID-19 pandemic in the Netherlands, a 46% decline in general practitioner consultations, and a sharp decrease in cancer diagnoses were reported [1, 2]. Increased (perceived) thresholds to health care access may negatively affect patients’ (psycho)social and physical well-being as well as their prognosis.

Symptoms of anxiety and depression among cancer patients and survivors have been reported to affect their health care behavior and health care consumption during a health threat of the magnitude of COVID-19 [3].

The aim of this study was to assess the prevalence of anxiety and depression among a large cohort of breast cancer patients and survivors during the COVID-19 pandemic. In addition, we assessed the association between the presence of symptoms of anxiety and/or depression and COVID-19-specific concerns, including health care behavior and consumption.

## Materials and methods

The present study was conducted within the prospective observational multicenter 'Utrecht cohort for Multiple BREast cancer intervention studies and Long-term evaLuAtion' (UMBRELLA), including patients with histologically proven invasive breast cancer or ductal carcinoma in situ (DCIS) referred from regional and tertiary referral hospitals to the University Medical Center Utrecht (UMCU) for adjuvant radiation therapy [4, 5]. All participants completed self-reported UMBRELLA questionnaires, including the Hospital Anxiety and Depression Scale (HADS), at regular time intervals during and after their breast cancer treatment.

All active participants, who enrolled in the UMBRELLA cohort between October 2013 and April 2020 and consented to receiving online questionnaires, were sent a survey (April 7, 2020), including the HADS and COVID-19-related questions [6, 7]. Ethical approval was obtained from the Medical Ethics Committee of the UMCU (NL52651.041.15, METC 15/165). The UMBRELLA study is registered on clinicaltrials.gov (NCT02839863).

HADS is a 14-item questionnaire using a 4-point Likert scale. Scores ≥ 12 on the total HADS, and scores ≥ 8 on the 7-item anxiety and depression subscales indicated increased risk of depression or anxiety disorder [8].

Demographic and clinical data, including age, highest educational level, type of surgery, most invasive axillary treatment, (neo-)adjuvant systemic treatment, radiotherapy, and pathological T and N stage (AJCC 7th edition), were collected in the context of UMBRELLA and provided by the Netherlands Cancer Registry (NKR) [9].

Frequencies, proportions, means with standard deviation, or medians with interquartile ranges were used to describe patient demographics, treatment characteristics and HADS scores. A Chi-square test was performed to compare differences in proportions of patients scoring above the clinically relevant HADS threshold scores before and during COVID-19 [10]. Univariable and multivariable logistic regression analyses were used to evaluate to what extent relevant clinical demographic, tumor- and treatment characteristics affected clinically relevant symptoms of anxiety and/or depression during COVID-19. Two-sided *p* values < 0.05 were considered statistically significant. Statistical analyses were performed with IBM Statistical Package for Social Sciences (SPSS) software, version 25 (IBM Corp, Armonk, NY).

## Results

Of the 3239 participants enrolled in the UMBRELLA cohort between October 2013 and April 2020, 1595 participants met the inclusion criteria. Of those, 1051 (66.0%) participants completed the COVID-19-specific questionnaire, of which 4.9% (*n* = 51) were under active treatment for their breast cancer.

### Prevalence and risk factors for anxiety and/or depression during COVID-19

Overall, 284 (27.0%) participants experienced clinically relevant symptoms of anxiety and/or depression during COVID-19 (Table 1). Of all participants who experienced symptoms of anxiety and/or depression during COVID-19, 62.7% (*n* = 156) already experienced these symptoms pre-COVID-19. A total of 18.2% (*n* = 191) of all participants reported symptoms of anxiety and 16.0% (*n* = 168) symptoms of depression during the pandemic. The proportion of participants experiencing symptoms of anxiety and/or depression was slightly, but significantly lower before the pandemic (23.4%, *n* = 220).

**Table 1:** Baseline characteristics of participants with or without a clinically relevant increase in total HADS score (≥ 12), i.e., symptoms of anxiety and/or depression, as measured by the HADS questionnaire during the COVID-19 pandemic (*n* = 1051)

	Participants with a total HADS score < 12^a^ (*n* = 767)		Participants with a total HADS score ≥ 12^a^ (*n* = 284)		*p* value
**Patient characteristics**					
Age in years at inclusion, mean (SD)	57	9.6	55	10.1	0.003
Sex, No. (%)					0.135
Female	761	99.2	284	100.0	
Male	6	0.8	0	0.0	
Highest educational level					0.185
Primary or (post-)secondary school	326	42.5	133	46.8	
College or university	434	56.6	147	51.8	
Unknown	7	0.9	4	1.4	
Time since diagnosis (months), median (IQR)	24	6–42	24	6–42	0.859
Unknown, No. (%)	6	0.8	3	1.1	
**Tumor characteristics**					
Pathological T stadium					0.698
In situ (IS), 0, I or II	698	91.0	256	90.1	
III or IV	15	2.0	8	2.8	
X or unknown	54	7.0	20	7.0	
Pathological N stadium					0.005
0	461	60.1	145	51.1	
I, II or III	198	25.8	102	35.9	
X or unknown	108	14.1	37	13.0	
**Treatment characteristics**					
Type of breast surgery					0.112
Breast conserving therapy	596	77.7	217	76.4	
Mastectomy	70	9.1	24	8.5	
Mastectomy with direct breast reconstruction	65	8.5	28	9.9	
No breast surgery	0	0.0	2	0.7	
Unknown	36	4.7	13	4.6	
Most invasive axillary treatment					0.509
Sentinel node procedure	572	74.6	212	74.6	
Axillary lymph node dissection^b^	56	7.3	26	9.2	
Unknown or not performed	139	18.1	46	16.2	
Systemic therapy^c^					0.008
No systemic therapy	168	21.9	38	13.4	
Chemotherapy^d^	261	34.0	117	41.2	
Endocrine therapy or immunotherapy	102	13.3	45	15.8	
Unknown	236	30.8	84	29.6	
Radiation therapy					0.003
Local	515	67.1	163	57.4	
Locoregional^e^	158	20.6	86	30.3	
No radiation therapy or type unknown	94	12.3	35	12.3	
Currently receiving active breast cancer treatment^f^					0.779
Yes	37	4.8	14	4.9	
No	723	94.3	266	93.7	
Other	7	0.9	4	1.4	
**Hospital Anxiety and Depression Scale**					
Total HADS score before COVID-19^a,g^					< 0.001
Above threshold	64	9.3	156	62.7	
Below threshold	627	90.7	93	37.3	
HADS anxiety score before COVID-19^a,g^					< 0.001
Above threshold	57	8.2	124	49.8	
Below threshold	634	91.8	125	50.2	
HADS depression score before COVID-19^a,g^					< 0.001
Above threshold	26	3.8	89	35.7	
Below threshold	665	96.2	160	64.3	

As a result of rounding, percentages may not total to 100%

*IQR* interquartile range*, n* number, *SD* standard deviation, *HADS* Hospital Anxiety and Depression Score

^a^A clinically relevant total HADS score ≥ 12 (above threshold) indicates a probable depression and/or anxiety disorder

^b^Axillary lymph node dissection (in combination with sentinel node procedure)

^c^Pre- and/or postoperative therapy

^d^Chemotherapy (in combination with other systemic therapy, i.e., immunotherapy, endocrine therapy)

^e^Including supraclavicular and/or axillary lymph nodes

^f^Active treatment includes chemotherapy and/or radiation therapy

^g^Only patients with known HADS scores as obtained in UMBRELLA within 2 years before the first official COVID-19 diagnosis in the Netherlands (February 27, 2020) were included for comparative analyses with HADS scores from the COVID-19-specific questionnaire (*n* = 940)

Lower age, higher pathological N stage, receipt of systemic therapy or radiotherapy and pre-existent symptoms of anxiety or depression were significantly associated with anxiety and/or depression during COVID-19 (Table 1). In multivariable analysis, only pre-existent symptoms of anxiety or depression were independently and significantly associated with symptoms of anxiety and/or depression during COVID-19 (OR 6.1, 95%CI 4.1–9.2 and OR 6.0, 95% CI 3.5–10.2, resp., Table 2).

**Table 2:** Multivariable logistic regression analysis of risk factors for clinically relevant increase in symptoms of anxiety and/or depression, i.e., total HADS score above threshold (≥ 12), during the COVID-19 pandemic (*n* = 940)

Variables	OR	95% CI
Age	0.99	0.97–1.01
Pathological N stadium		
0	Ref.	
I, II or III	1.12	0.67–1.85
X or unknown	1.36	0.75–2.45
Systemic therapy^a^		
No systemic therapy	Ref.	
Chemotherapy^b^	1.48	0.85–2.55
Endocrine therapy or immunotherapy	1.55	0.83–2.88
Unknown	1.41	0.81–2.44
Radiation therapy		
Local	Ref.	
Locoregional^c^	1.49	0.89–2.51
No radiation therapy or type unknown	0.83	0.45–1.55
HADS anxiety score before COVID-19^a^		
Below threshold	Ref.	
Above threshold	6.12	4.05–9.24
HADS depression score before COVID-19^a^		
Below threshold	Ref.	
Above threshold	5.95	3.48–10.18

^a^Pre- and/or postoperative therapy

^b^Chemotherapy (in combination with other systemic therapy, i.e., immunotherapy, endocrine therapy)

^c^Including supraclavicular and/or axillary lymph nodes

### COVID-19-specific concerns and health care consumption

Significantly more participants with anxiety and/or depression experienced higher barriers to contact their general practitioner (47.5% vs. 25.0%, resp.) and breast cancer physicians (26.8% vs. 11.2%, resp.) compared to patients without these symptoms (Table 3). In addition, a higher proportion of participants with anxiety and/or depression reported that their current treatment or (after)care was affected by COVID-19 compared to those without these symptoms (32.7% vs. 20.5%, resp.).

**Table 3:** Patient-reported outcomes of COVID-19-specific concerns in the early months of the COVID-19 pandemic in the Netherlands (*n* = 1051)

	Participants with a total HADS score < 12 during COVID-19^a^		Participants with a total HADS score ≥ 12 during COVID-19^a^		*p* value
	No.	%	No.	%	
Are/were you infected by the COVID-19?					0.368
Yes, confirmed by nasopharyngeal swab	1	0.1	0	0.0	
Possibly, I have or had fever	66	8.6	34	12.0	
No, I was tested negative	7	0.9	2	0.7	
No, I had/have no symptoms and I was not tested	693	90.4	248	87.3	
Is the current COVID-19 measure affecting your current treatment or aftercare?					< 0.001
Yes	184	24.0	102	35.9	
No	583	76.0	182	64.1	
Do you expect that the current COVID-19 measures will affect your treatment or aftercare in the future?					< 0.001
Yes	157	20.5	93	32.7	
No	610	79.5	191	67.3	
Did the threshold to contact your general practitioner change, because of the COVID-19 situation?					< 0.001
Yes, I contact my general practitioner more easily	13	1.7	6	2.1	
Yes, I contact my general practitioner less easily	192	25.0	135	47.5	
No	562	73.3	143	50.4	
Did the threshold to contact the physicians treating your breast cancer change, because of the COVID-19 situation?					< 0.001
Yes, I contact my breast cancer physicians more easily	7	0.9	1	0.4	
Yes, I contact my breast cancer physicians less easily	86	11.2	76	26.8	
No	674	87.9	207	72.9	

^a^A clinically relevant total HADS score ≥ 12 (above threshold) indicates a probable depression and/or anxiety disorder

## Discussion

During the COVID-19 pandemic, 27.0% of the (ex-)breast cancer patients reported clinically relevant symptoms of anxiety and/or depression. (Ex-)breast cancer patients with anxiety and/or depression reported to experience higher thresholds to contact their health care providers. Factors independently associated with anxiety and/or depression during COVID-19 were pre-existent symptoms of anxiety or depression.

In the early months of the COVID-19 pandemic, a sharp decrease in cancer diagnoses and a 46% decline in general practitioner consultations were observed in the Netherlands [1, 2]. In this study, participants with anxiety and/or depression reported higher barriers to contact their health care providers during COVID-19 compared to those without these symptoms. The COVID-19 pandemic might have increased the perceived burden on patients with symptoms of anxiety and/or depression. High levels of anxiety and perceived threat have been shown to be related to increased avoidance behavior [11]. Anxiety for a COVID-19 infection, a higher level of moral concerns about wasting the physicians’ time for non-COVID-19-related symptoms, and assumptions about scarcity in the capacity of health care services for non-COVID-19-related care might also explain these barriers in seeking health care among patients with symptoms of anxiety and/or depression [1]. These results suggest that, in case of subsequent waves or a future pandemic, (ex-)breast cancer patients experiencing anxiety and/or depression are especially at risk for reduced health care consumption and may need extra encouragement to contact their health care providers, when necessary.

The proportion of participants experiencing anxiety and/or depression increased only slightly during COVID-19 (27.0%), when compared to pre-COVID-19 (23.4%). However, there was a considerable shift: quite some patients with pre-existing symptoms of anxiety and/or depression no longer experienced clinically relevant levels of anxiety and/or depression during COVID-19 and vice versa. The participants’ perception of the COVID-19 pandemic and different coping mechanisms might play a role in this shift [11]. Higher tolerance for uncertainty is related to better coping strategies and lower threat appraisal. For example, patients who tolerated uncertainty better, experienced lower levels of anxiety during the H1N1 pandemic in 2009 and showed higher levels of problem-focused coping [11]. However, further studies are needed to better understand these coping mechanisms in the context of a major health threat, and their effect on health care consumption.

Our findings that 18.2% of all participants reported symptoms of anxiety and 16.0% symptoms of depression, are in line with outcomes previously reported among UMBRELLA participants with early invasive breast cancer or DCIS prior to COVID-19 at 24 months after diagnosis (anxiety 12.5–17.1%, depression 6.0–16.1%) [12]. Moreover, the observed proportion of (ex-)breast cancer patients with anxiety and/or depression during COVID-19 seemed even lower when compared to the general population. A recent meta-analysis assessing anxiety and depression among a general population in Europe during COVID-19 observed that 32.4% (*n* = 8.341) experienced symptoms of anxiety and 23.8% (*n* = 8.341) symptoms of depression [13].

Although previous research showed that mental health can be impaired in the context of a major health threat, there are still insufficient clinical (screening) tools that can help identify those at risk [14]. Pre-existent anxiety and/or depression was reported by 62.7% (*n* = 156) of the participants with anxiety and/or depression during COVID-19. Similarly, a recent global study among non-cancer patients with pre-existent anxiety or depression (*n* = 2.734) reported worsening of psychological well-being in at least 50.0% during COVID-19 [15].

With the aim of providing adequate (mental) health support in the context of a health threat of this magnitude, surveillance and clear documentation of symptoms of anxiety and/or depression prior to and during major health threats seem a valuable tool to identify those at risk for reduced health care consumption. Especially in times of lockdown and social distancing, integration of e-mental health applications and digital psychological interventions is important to improve supportive care for those at risk. Last, raising public awareness about the importance of encouraging these particularly vulnerable individuals to contact their health care professionals, when necessary, is warranted.

## Conclusion

Reduced health care consumption, and increased thresholds to contact health care professionals following the COVID-19 pandemic are reasons for concern. We found that (ex-)breast cancer patients with symptoms of anxiety and/or depression experienced higher barriers in seeking health care. Patients with pre-existent symptoms of anxiety or depression were particularly at risk for (worsening of) anxiety and/or depression during the pandemic.

## Data Availability

Raw data were generated at the University Medical Center Utrecht. Derived data supporting the findings of this study are available from the corresponding author Prof Dr HM Verkooijen upon reasonable request.

## Abbreviations

CI: 95% Confidence interval
COVID-19: Coronavirus disease 2019
DCIS: Ductal carcinoma in situ
EORTC: European Organization for Research and Treatment of Cancer
HADS: Hospital Anxiety and Depression Scale
IQR: Interquartile range
n: Number
NKR: Netherlands Cancer Registry
OR: Odds ratio
PRO(s): Patient-reported outcome(s)
SD: Standard deviation
SPSS: Statistical package for social sciences
UMBRELLA: Utrecht cohort for Multiple BREast cancer intervention studies and Long-term evaluation
UMCU: University Medical Center Utrecht
